# Prognostic and Functional Significance of MAP4K5 in Pancreatic Cancer

**DOI:** 10.1371/journal.pone.0152300

**Published:** 2016-03-29

**Authors:** Oliver H. Wang, Nancy Azizian, Ming Guo, Michela Capello, Defeng Deng, Fenglin Zang, Jason Fry, Matthew H. Katz, Jason B. Fleming, Jeffrey E. Lee, Robert A. Wolff, Samir Hanash, Huamin Wang, Anirban Maitra

**Affiliations:** 1 Department of Pathology, The University of Texas M. D. Anderson Cancer Center, Houston, Texas, United States of America; 2 Department of Clinical Cancer Prevention, The University of Texas M. D. Anderson Cancer Center, Houston, Texas, United States of America; 3 Department of Cancer Biology, The University of Texas M. D. Anderson Cancer Center, Houston, Texas, United States of America; 4 Department of Surgical Oncology, The University of Texas M. D. Anderson Cancer Center, Houston, Texas, United States of America; 5 Department of Gastrointestinal Medical Oncology, The University of Texas M. D. Anderson Cancer Center, Houston, Texas, United States of America; 6 Department of Translational Molecular Pathology, The University of Texas M. D. Anderson Cancer Center, Houston, Texas, United States of America; Indiana University School of Medicine, UNITED STATES

## Abstract

**Objectives:**

MAP4K5 plays an important role in regulating a range of cellular responses and is involved in Wnt signaling in hematopoietic cells. However, its functions in human malignancies have not been studied. The major objectives of this study are to examine the expression, functions and clinical significance of MAP4K5 in pancreatic ductal adenocarcinoma (PDAC).

**Materials and Methods:**

The expression levels of MAP4K5, E-cadherin, vimentin, and carboxylesterase 2 (CES2) were examined by immunohistochemistry in 105 PDAC and matched non-neoplastic pancreas samples from our institution. The RNA sequencing data of 112 PDAC patients were downloaded from the TCGA data portal. Immunoblotting and RNA sequencing analysis were used to examine the expression of MAP4K5 and E-cadherin in pancreatic cancer cell lines. The effect of knockdown MAP4K5 using siRNA on the expression of CDH1 and vimentin were examined by Real-time RT-PCR in Panc-1 and AsPC-1 cells. Statistical analyses were performed using IBM SPSS Statistics.

**Results:**

MAP4K5 protein is expressed at high levels specifically in the pancreatic ductal cells of 100% non-neoplastic pancreas samples, but is decreased or lost in 77.1% (81/105) of PDAC samples. MAP4K5-low correlated with the loss of E-cadherin (P = 0.001) and reduced CES2 expression (P = 0.002) in our patient populations. The expression levels of MAP4K5 mRNA directly correlated with the expression levels of CDH1 mRNA (R = 0.2490, P = 0.008) in the second cohort of 112 PDAC patients from The Cancer Genome Atlas (TCGA) RNA-seq dataset. Similar correlations between the expression of MAP4K5 and E-cadherin were observed both at protein and mRNA levels in multiple pancreatic cancer cell lines. Knockdown MAP4K5 led to decreased CDH1 mRNA expression in Panc-1 and AsPC-1 cells. MAP4K5-low correlated significantly with reduced overall survival and was an independent prognosticator in patients with stage II PDAC.

**Conclusions:**

MAP4K5 expression is decreased or lost in majority of PDACs. The strong associations between low MAP4K5 expression and loss of E-cadherin, reduced CES2 expression and decreased overall survival may suggest an important role of MAP4K5 in epithelial-to-mesenchymal transition, chemotherapy resistance and tumor progression in pancreatic cancer. Targeting impaired MAP4K5 signaling may represent a new therapeutic strategy for pancreatic cancer.

## Introduction

Pancreatic cancer is one of the most lethal diseases among all human malignancies with only 6% of patients alive 5 years after diagnosis [1]. Currently, pancreatic cancer is the fourth leading cause of cancer-related death in the United States [2]. Due to the lack of early detection, effective treatment, and prevention for pancreatic cancer, it has been predicted that pancreatic cancer will become the second leading cause of cancer-related death in the United States by 2030 [3].

Pancreatic cancer is well-known for its sub-optimal responses to conventional chemotherapies and radiation therapies. Overcoming drug resistance has been one of the major obstacles to improving the clinical outcomes for patients with pancreatic cancer [4, 5]. It has been shown that epithelial-mesenchymal transition (EMT) contributes, at least in part, to the therapeutic resistance of pancreatic cancer [5, 6]. EMT plays important role during embryogenesis, wound healing and tumor progression and is characterized by the loss of epithelial marker E-cadherin and gain expression of mesenchymal markers such as N-cadherin, vimentin and fibronectin etc. Repression of epithelial phenotype and gain of mesenchymal phenotype are regulated by many molecules, including master transcription factor regulators, Snail, Slug, Twist, and Zeb-1 [7, 8]. The molecular mechanisms of EMT regulation in pancreatic cancer remain unclear.

The mitogen-activated protein (MAP) kinase pathway plays an important role in mutant Kras-driven pancreatic tumorigenesis and is involved in the regulation of EMT through transforming growth factor beta (TGFβ) [9]. MAP4K5, also named germinal center kinase-related (GCKR) or kinase homologous to STE20 (KHS1), belongs to the mammalian Ste20-like serine/threonine kinase family. Within this kinase family, both HPK1 (also named MAP4K1) and MAP4K5 are grouped in the germinal center kinase subfamily and function as MAP4Ks in that they activate MAP3Ks, which signal through the well-established MKK4/MKK7, c-Jun N-terminal kinase (JNK), or stress-activated protein kinase (SAPK) cascades [10, 11]. Recent studies showed that HPK1 may function as a novel tumor suppressor in pancreatic cancer. The progressive loss of HPK1 protein mediated by the 26S proteasome is strongly associated with the progression from early pancreatic intraepithelial neoplasia to invasive pancreatic ductal adenocarcinoma (PDAC). Restoration of HPK1 protein expression in PDAC cells either by proteasome inhibitor or knockdown Fbxw8 causes cell cycle arrest and inhibits PDAC cell growth, which are mediated through stabilization of p21 and p27 [12, 13]. On the other hand, MAP4K4 is overexpressed in most human cancer cells including pancreatic cancer and promotes tumor cell migration and invasion [14–16]. Overexpression of MAP4K4 is associated with poor prognosis in patients with stage II PDAC after pancreaticoduodenectomy [16]. These studies suggest the functional importance of MAP4K proteins in pancreatic cancer. However, the expression and functions of MAP4K5 in cancer have not been examined. In this study, we examined the expression of MAP4K5 by immunohistochemistry in PDAC and matched non-neoplastic pancreas samples from 105 patients who underwent pancreaticoduodenectomy at our institution. We correlated the results of MAP4K5 expression with clinicopathologic features, overall survival, EMT markers (E-cadherin, Vimentin) and carboxylesterase 2 (CES2). We validated our findings in another independent cohort of 112 PDAC patients from The Cancer Genome Atlas (TCGA) database and in human PDAC cell lines. In addition, we examined the effects of MAP4K5 knockdown using siRNA on the expression of EMT markers in PDAC cell lines. Our results may suggest that loss of MAP4K5 expression plays a role in EMT, chemotherapy resistance and progression of pancreatic cancer. Thus targeting impaired MAP4K5 signaling pathways may help to overcome the drug resistance of pancreatic cancer.

## Materials and Methods

### Patient population

Our study population consisted of 105 patients (57 male and 48 female) with stage II PDAC who underwent pancreas resection with curative intent at our institution. Patient age ranged from 24.9 to 84.8 years (median age: 64.2 years). Pancreaticoduodenectomy, distal pancreatectomy, and total pancreatectomy were performed in 86, 17 and 2 patients, respectively. No patients received pre-operative neoadjuvant chemotherapy and/or radiation therapy. Patients who underwent pancreatectomy for carcinomas of the duodenum, ampulla of Vater, common bile duct or other types of pancreatic neoplasms were excluded. Three patients with stage IV disease were also excluded because the numbers of cases for stage IV disease was too small to be representative. Written consents from all patients were obtained. This study and the immunnohistochemistry were approved by the Institutional Review Board of the University of Texas M.D. Anderson Cancer Center.

### Tissue microarray construction

The pancreatic cancer tissue microarrays were constructed as previously described using a tissue microarrayer (Beecher Instruments, Sun Prairie, WI)[17]. Briefly, representative areas of tumor and matched non-neoplastic pancreatic tissue were selected based on the review of hematoxylin and eosin (H & E) stained slides. The corresponding formalin-fixed paraffin embedded tissue blocks were retrieved. For each patient, two 1.0 mm cores from the representative areas of the tumor and one 1.0 mm core of the matched non-neoplastic pancreas were used for tissue microarray construction.

### Immunohistochemistry

Immunohistochemical stains were performed on 5.0 μm unstained sections from tissue microarray blocks as previously reported [18, 19] using the following antibodies: MAP4K5 (ab56848, Cambridge, MA, 1:400 dilution); E-cadherin (Life Technologies, clone HECD-1, Camarillo, CA, 1:7000 dilution); vimentin (DAKO clone V9, Carpeninteria, CA, 1:900 dilution) and anti-carboxylesterase 2 (CES2, Sigma Aldrich, HPA018897, St. Louis, MO, 1:1000 dilution). In brief, antigen retrieval was performed by treatment in a steamer for 25 min, followed by a standard indirect immunoperoxidase procedure (ABC-Elite, Vector lab, Burlingame, CA). Immunohistochemical stained slides were evaluated independently by two pathologists (M.G. and H.W.). Based on the staining intensity, the expression of MAP4K5 was categorized as MAP4K5-low (negative or weak cytoplasmic staining in tumor cells) or MAP4K5-high (moderate or strong cytoplasmic staining in tumor cells) since all the tumors were either diffusely positive or negative for MAP4K5. The expression of E-cadherin was categorized as E-cadherin-high (≥50% tumor cells with membranous staining) or E-cadherin-low (<50% of tumor cells with membranous staining) as previously described [6, 20]. The expression of vimentin was categorized as positive if cytoplasmic staining for vimentin was detected in any cancer cells or negative if the tumor showed complete negative staining for vimentin as previously reported [20]. The expression of CES2 was classified as CES2-low and CES2-high using the criteria as previous described [21].

### The Cancer Genome Atlas (TCGA) RNA sequencing data analysis

Level 3 RNA sequencing (RNA-seq) data generated using the Illumina HiSeq 2000 RNA Sequencing Version 2 platform of 112 pancreatic ductal adenocarcinoma patients were downloaded from the TCGA data portal (https://tcga-data.nci.nih.gov). The individual RNA-seq sample expression files were compiled into a dataset using R v3.1.1 and R-studio v0.98.1062. Gene expression level was determined using RSEM normalized read counts.

### Cell culture and immunoblotting

Human pancreatic cancer cell lines MIA PaCa-2, Panc-1, AsPC-1, CFPAC-1, Panc-3, L3.6PL, MPanc-96, BxPC-3, SW1990, SU.86.86, Hs 766T, Panc 03.27, HPAF-II and Capan-2 were purchased from the American Type Culture Collection (Manassas, VA) and were cultured either in Dulbecco’s modified Eagle’s medium or in RPMI-1640 medium supplemented with 10% fetal bovine serum in a humidified incubator containing 5% CO_2_ at 37°C. The HPDE cell line, an immortalized human pancreatic ductal cell line, was a generous gift from Dr. Ming-Sound Tsao [22]. The proteins in the whole cell lysates were separated by 10% SDS-PAGE, electroblotted onto PVDF membranes (Novex, San Diego, CA), blocked in 5% skim milk in 1x TBS, and probed with the following primary antibodies against MAP4K5, E-cadherin, and actin. The 293T cells transfected with MAP4K5 cDNA construct saved as a positive control for MAP4K5 immunoblotting. Proteins were detected using an enhanced chemiluminescence (ECL) kit (Amersham-Pharmacia Biotech, Piscataway, NJ).

### RNA sequencing analysis

RNA extraction was performed using RNeasy Maxi Kit (Qiagen) from 11 human pancreatic cancer cell lines (BxPC-3, SW1990, SU.86.86, Panc-1, Hs 766T, CFPAC-1, MIA PaCa-2, AsPC-1, Panc 03.27, HPAF-II and Capan-2). RNA-Seq libraries were prepared by using standard Illumina reagents and protocols. Paired-end sequencing with the read length of 50 bases were performed on the Illumina HiSeq 2000 platform following the manufacturer’s instructions. CASAVA 1.8.2 was used to demultiplex and to generate FASTQ files. The sequencing data were analyzed using the software packages of Tophat (Trapnell C, et al. Bioinformatics 2009) mapping against hg19 and Cufflinks (Trapnell C, et al. Nat Biotechnol 2010). The number of reads was normalized and expressed as fragments per kilobase of exon per million fragments mapped [23].

### Knockdown of MAP4K5 expression using siRNA and Real-time RT-PCR analysis

Panc-1 or AsPC-1 cells were plated on 100-mm dishes and transiently transfected with Dharmacon SMARTpool control and MAP4K5 siRNA at final concentrations of 25 nmol/L using Hiperfect transfection reagent (QIAGEN) according to the manufacturer's protocol. Cells were incubated with the siRNA complex for 48 h, then harvested for real-time reverse transcription-PCR (RT-PCR) as described previously [6]. In brief, RNA was prepared using TRIzol (Invitrogen) according to the manufacturer's instructions. The quality of RNA was evaluated using spectrophotometry. The cDNA used for subsequent PCR was made using iScript (Bio-Rad Laboratories) and SensiMix real-time PCR kit was used for real-time PCR (Bioline, Taunton, MA) for MAP4K5, CDH1 and vimentin. The 18s rRNA was used as an internal reference gene to normalize the input cDNA.

### Statistical analyses

Statistical analyses were performed using IBM SPSS Statistics (version 22, IBM SPSS Inc., Chicago, IL). Categorical data of MAP4K5 expression and correlations with clinicopathologic features and the expression of E-cadherin, vimentin and CSE2 were analyzed using Chi-square and Fisher’s exact tests. Overall survival curves were constructed using a Kaplan-Meier method. The statistical significance of differences in survival was analyzed using the log-rank tests. Univariate and multivariate Cox-regression analyses via a backward stepwise procedure were performed. A P-value of <0.05 were considered statistically significant.

## Results

### Expression of MAP4K5 is decreased in PDAC samples

**Fig 1:**
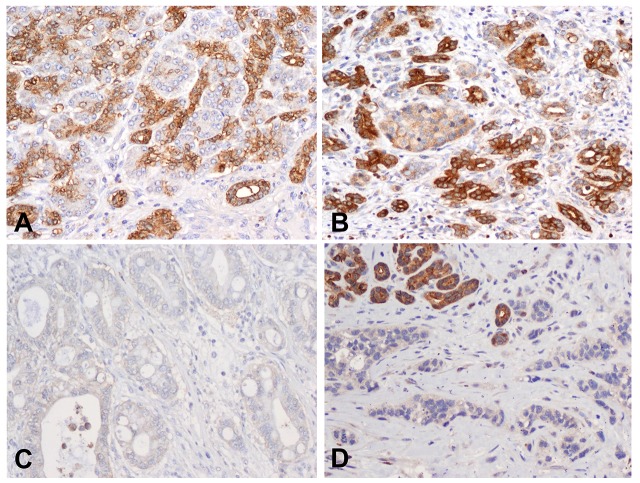
Expression of MAP4K5 in non-neoplastic pancreas and pancreatic ductal adenocarcinoma samples. Representative micrographs show the expression of MAP4K5 in normal pancreatic tissue (A) and chronic pancreatitis (B). The benign pancreatic ductal cells in normal pancreas and chronic pancreatitis show strong cytoplasmic staining for MAP4K5. The adjacent pancreatic acinar cells and pancreatic islet cells are either negative or have very low expression of MAP4K5. C & D, Representative micrographs show the loss of MAP4K5 expression in two different pancreatic ductal adenocarcinoma samples. Strong cytoplasmic staining for MAP4K5 in benign pancreatic ductal cells in the left upper corner in D served as internal positive control. Original magnifications: 200X.

Immunohistochemical staining for MAP4K5 showed diffuse cytoplasmic staining specifically in the pancreatic ductal cells of non-neoplastic pancreas samples. The pancreatic acinar cells and islet cells had no or very low expression of MAP4K5 (Fig 1A and 1B). In PDAC cases that were MAP4K5 positive, diffuse cytoplasmic staining were observed only carcinoma cells. High level of MAP4K5 expression was detected in the normal pancreatic ductal cells of all matched non-neoplastic pancreas samples (100%). In contrast, among the 105 PDAC samples, only 24 (22.9%) were MAP4K5-high and 81 (77.1%) tumors had no or very low expression of MAP4K5 (MAP4K5-low, Fig 1C and 1D). The expression of MAP4K5 was higher in normal pancreatic ductal cells than PDAC (Table 1, P = 0.0001). These data suggest that loss of MAP4K5 expression may be used as a diagnostic marker for PDAC.

**Table 1:** Expression of MAP4K5 in pancreatic ductal adenocarcinoma and benign pancreas samples.

	MAP4K5-high	MAP4K5-low	Total
Benign pancreas	105 (100%)	0 (0%)	105
PDAC	24 (22.9%)	81 (77.1%)	105

### The expression levels of MAP4K5 correlate with expression of E-cadherin in human PDAC samples in two independent cohorts of patients

**Fig 2:**
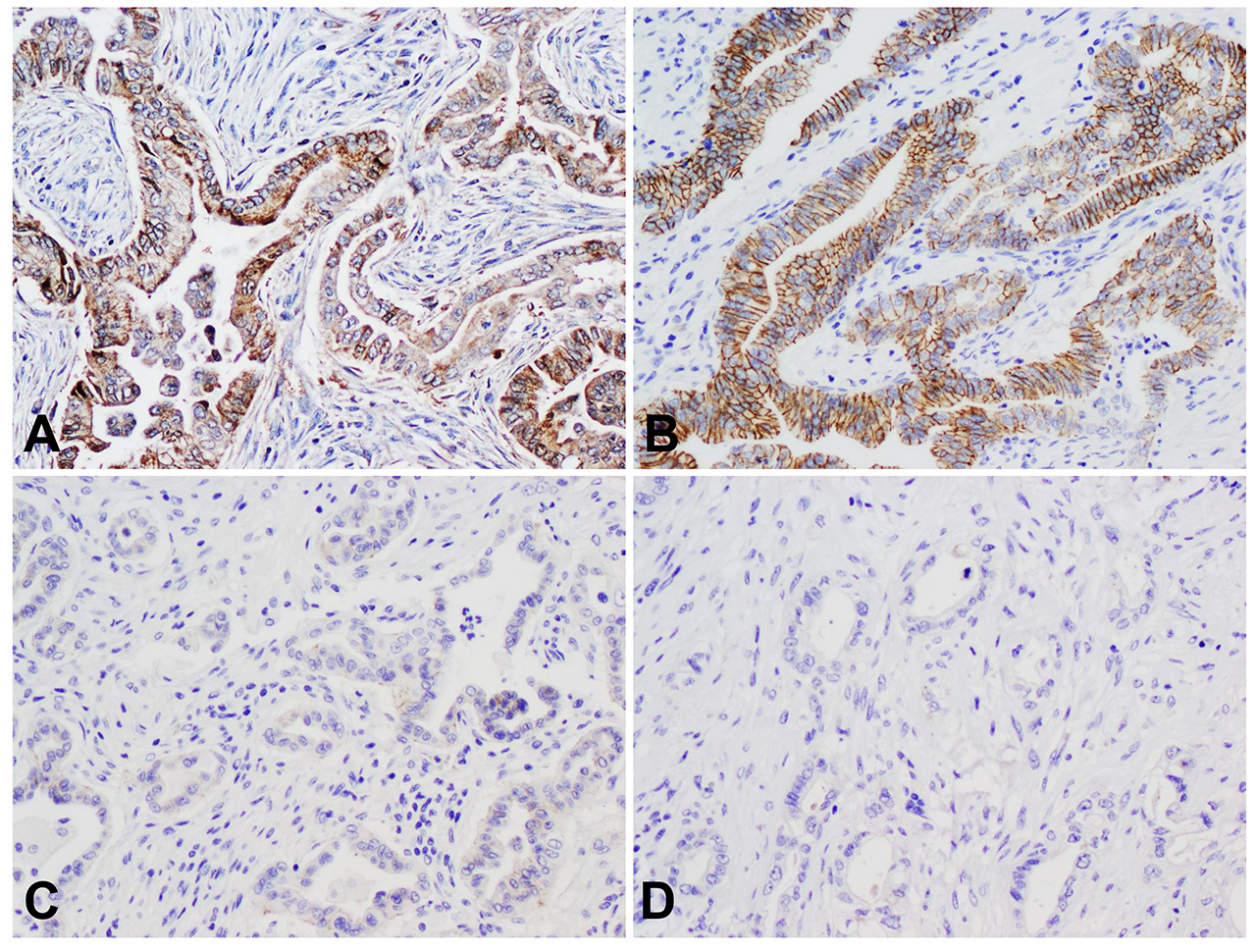
Expression of MAP4K5 correlates with the expression of E-cadherin in pancreatic ductal adenocarcinomas. A & B, Representative micrographs show strong cytoplasmic staining for MAP4K5 and strong membranous staining for E-cadherin in a pancreatic ductal adenocarcinoma. C & D, Representative micrographs show loss of MAP4K5 expression and the loss of E-cadherin expression in a pancreatic ductal adenocarcinoma. Original magnifications: 200X.

Representative micrographs showing the correlation between MAP4K5 expression and the expression of E-cadherin in PDAC samples are shown in Fig 2A–2D. We found that loss of MAP4K5 expression (MAP4K5-low) correlated with the loss of E-cadherin expression in PDAC in our patient population. Among the 24 cases that were MAP4K5-high, only 1 (4.2%) had low expression of E-cadherin. In contrast, 31 of 81 (38.3%) tumors that were MAP4K5-low had low expression of E-cadherin (P = 0.001, Table 2 and Fig 2A–2D). We also observed an increase in vimentin expression in MAP4K5-low tumors in our patient population. However, the difference in vimentin expression between the MAP4K5-low and MAP4K5-high group was not statistically significant (P = 0.30).

**Table 2:** Correlations of MAP4K5 Expression with Clinicopathologic Parameters And Other Molecular Markers in Pancreatic Cancer.

	MAP4K5-high	MAP4K5-low	P value
Gender			0.63
Female	12	36	
Male	12	45	
Average age ± SD, years	64.7±10.1	64.1±10.4	0.83
Average tumor size ± SD, cm	3.58±2.20	3.32±1.44	0.49
Tumor size			0.41
≤2.0 cm	2	12	
>2.0 cm	22	69	
Differentiation			0.17
Well to moderate	20	56	
Poor	4	25	
Nodal metastasis			0.74
Positive	20	65	
Negative	4	16	
Margin status			0.28
Positive	2	14	
Negative	22	67	
Recurrence			0.65
No recurrence	6	19	
Local	3	17	
Distant	15	45	
E-cadherin			0.001
High	23	50	
Low	1	31	
Vimentin			0.30
Positive	3	18	
Negative	21	63	
CES2*			0.002
High	20	39	
Low	3	37	

*CES2 expression data were available in 99 patients.

**Fig 3:**
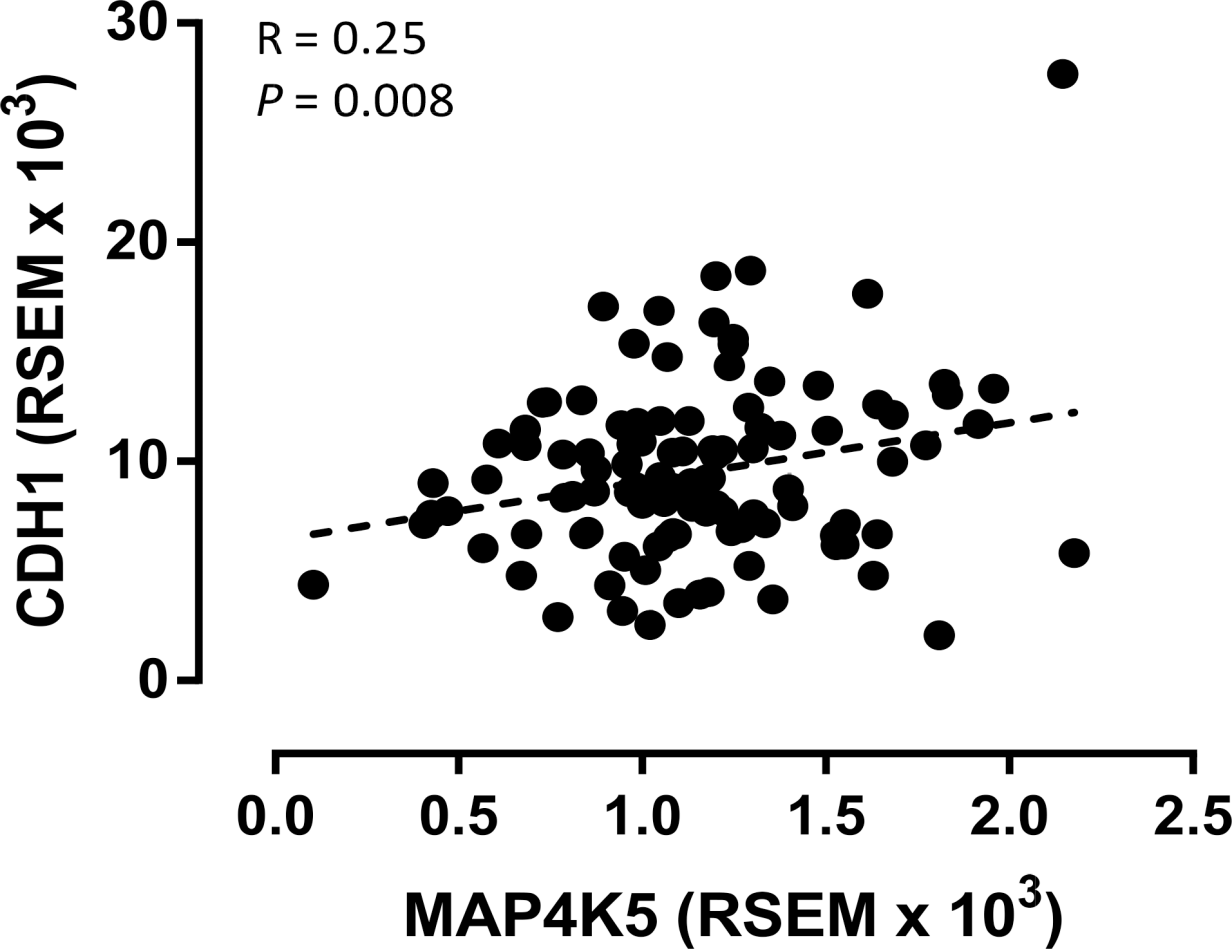
Pearson’s correlation analysis between MAP4K5 and CDH1 mRNA expression measured by RNA sequencing analysis in 112 pancreatic ductal adenocarcinoma samples from The Cancer Genome Atlas (TCGA) data portal.

To validate the correlation between the expression of MAP4K5 and E-Cadherin identified in our patient population, we examined the correlation between the expression levels of MAP4K5 mRNA and CDH1 mRNA in 112 PDAC patients using the RNA sequencing data downloaded from the TCGA data portal. We found that the mRNA levels of MAP4K5 directly correlated with the expression levels of CDH1 mRNA (R = 0.2490, p = 0.008, Fig 3) in TCGA patient population. The mRNA level of CDH1 was inversely correlated with the expression of vimentin mRNA in this patient population (R = -0.3496, p = 0.0002, data not shown).

### The expression levels of MAP4K5 correlate with the expression of E-cadherin in human PDAC cell lines

**Fig 4:**
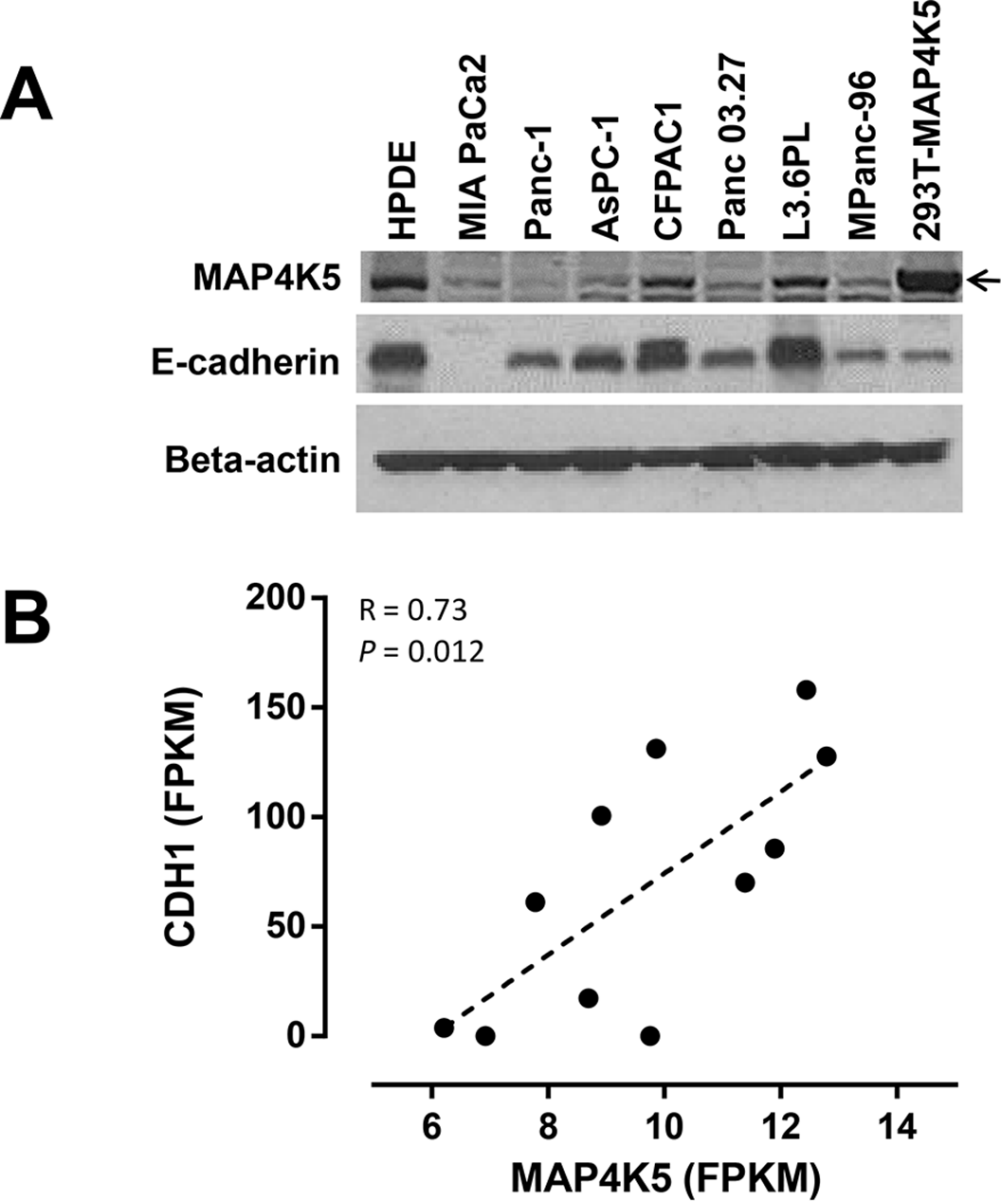
A. Immunoblotting for MAP4K5 and E-cadherin expression in HPDE cells, immortalized human pancreatic ductal cells, and pancreatic cancer cell lines. The 293T cells transfected with MAP4K5 cDNA construct saved as a positive control for immunoblotting. B. Pearson’s correlation analysis between the MAP4K5 mRNA and CDH1 mRNA expression by RNA-sequencing analysis in 11 pancreatic cancer cell lines.

Consistent with our immunohistochemical staining results, high level of MAP4K5 protein expression was detected in HPDE cells, immortalized normal pancreatic ductal cells, but only in 2 of 7 (28.6%) human pancreatic cancer cell lines by immunoblotting analysis (Fig 4A). To further confirm the correlation between the expression of MAP4K5 and E-cadherin observed in PDAC patients, we measured the expression levels of MAP4K5 and E-cadherin in seven PDAC cell lines by immunoblotting and found a similar correlation between the loss of MAP4A5 and E-cadherin protein expression in these PDAC cell lines. The HPDE, CFPAC1 and L3.6PL cells that expressed high levels of MAP4K5 also showed high levels of E-cadherin expression. Compared to these cells, all other pancreatic cancer cell lines that had low MAP4K5 expression had decreased expression of E-cadherin (Fig 4A). In addition, we performed RNA sequencing analysis of eleven PDAC cell lines and found a significant direct correlation between the expression levels of MAP4K5 mRNA and CDH1 mRNA (R = 0.7248, p = 0.012, Fig 4B) which is similar to the results from the TCGA patient population. The expression of CDH1 mRNA was indirectly correlated with mRNA levels of vimentin in these cell lines (R = -0.6087, p = 0.047, data not shown).

### Knockdown MAP4K5 expression leads to decreased E-cadherin in PDAC cell lines

**Fig 5:**
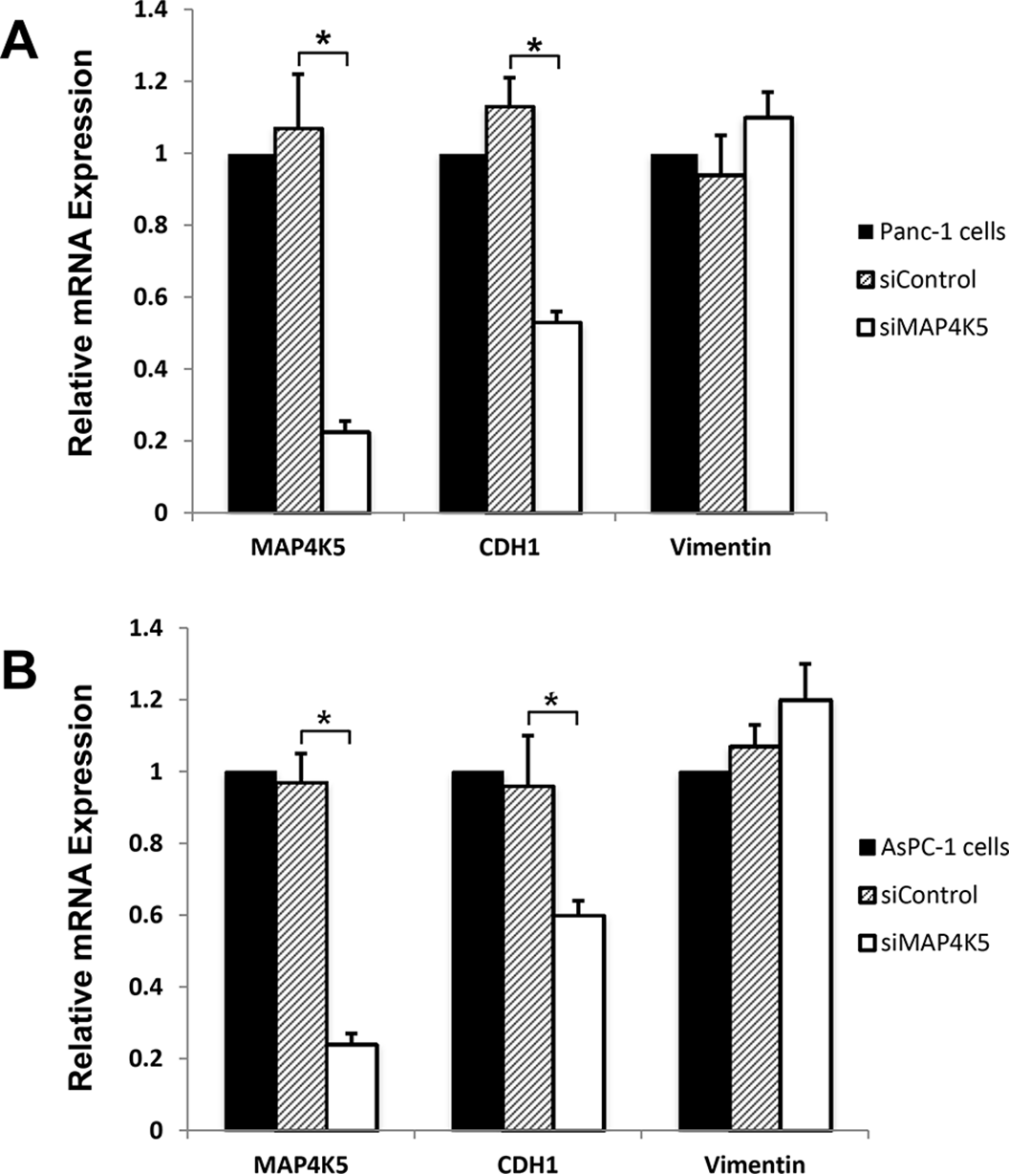
Knockdown MAP4K5 expression leads to decreased expression of E-cadherin mRNA, but no significant changes in the expression of vimentin mRNA in Panc-1 (A) and AsPC-1 cells (B). *P<0.01.

To evaluate the effects of MAP4K5 on CDH1 gene expression, we transiently transfected Panc-1 and AsPC-1 cells with siControl or siMAP4K5 and measured the mRNA levels of CDH1 and vimentin by quantitative RT-PCR. We found that silencing of MAP4K5 led to a significant decrease in the expression levels of CDH1 mRNA in both cell lines, but not the mRNA levels of vimentin (Fig 5A and 5B). Our data suggest that MAP4K5 may play a role in regulating the expression of CDH1 gene and EMT phenotype in PDAC.

### Loss of MAP4K5 expression correlates with CES2 expression in human PDAC samples

In the light of recent report that CES2, the most efficient carboxyl esterase in activating the prodrug irinotecan into the active metabolite SN-38, predicted the tumor response to irinotecan and neoadjuvant FOLFIRINOX therapy in patients with PDAC [21], we examined the correlation between the loss of MAP4K5 expression and CES2 expression in our patient population. We found that loss of MAP4K5 expression correlated significantly with low expression of CES2. Low CES2 expression was present in 48.7% MAP4K5-low tumors compared to 13.0% in MAP4K5-high tumors (P = 0.002, Table 2). No significant correlation between the expression of MAP4K5 and CES2 mRNA was observed in the TCGA patient population.

### Low MAP4K5 expression is associated with shorter overall survival

**Fig 6:**
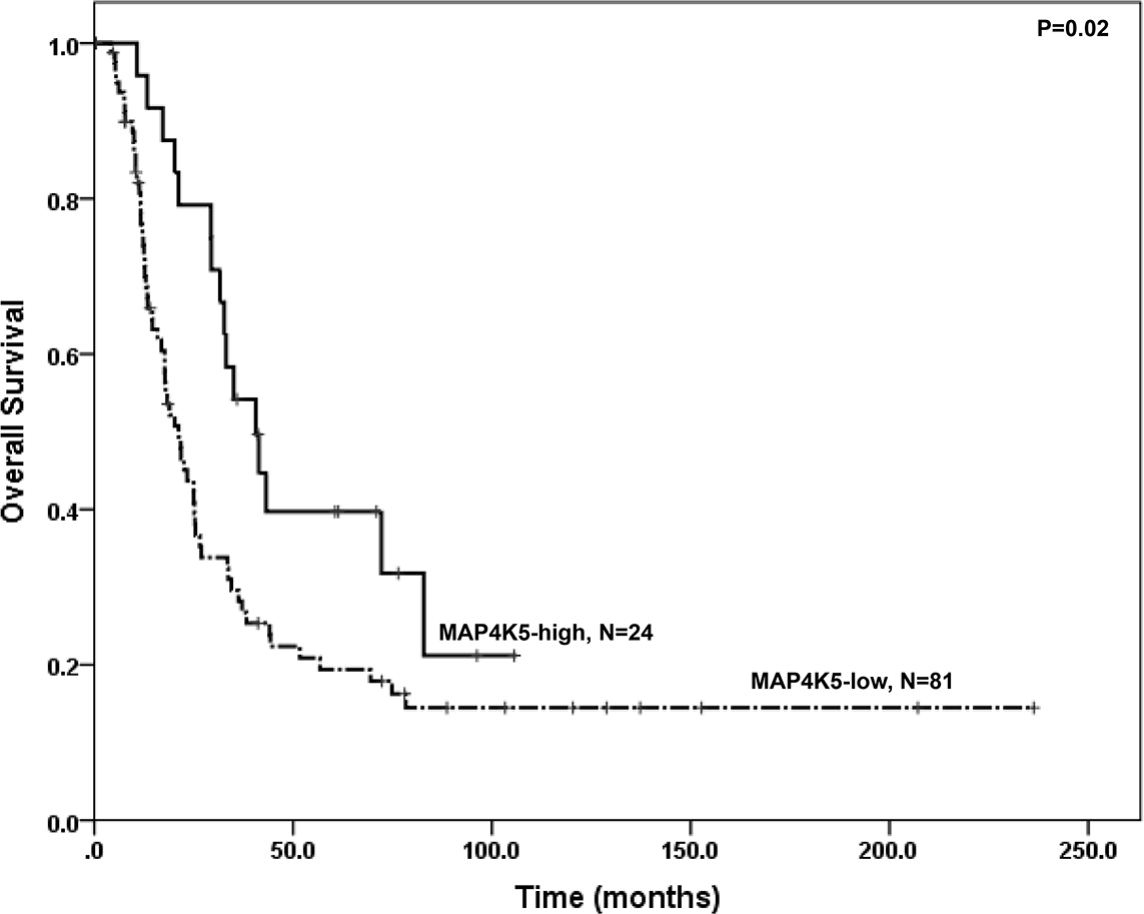
Kaplan-Meier curves for overall survival stratified by MAP4K5 protein expression in patients with stage II pancreatic ductal adenocarcinoma. Patients whose tumors had no or low MAP4K5 expression (MAP4K5-low) had shorter overall survival than patients whose tumors were MAP4K5-high.

The median follow up time after diagnosis of PDAC was 21.8 months in our patient population (range: 4.2 to 236.3 months). The median survival was 21.2 ± 2.6 months in MAP4K5-low group compared to 40.6 ± 6.2 months in patients whose tumor was MAP4K5-high (P = 0.02, log-rank test, Fig 6). Loss of MAP4K5 expression in PDAC correlated significantly with reduced overall survival. However, we did not observe significant correlation between the expression of E-cadherin, vimentin, or CES2 and survival (P>0.05, data not shown). In multivariate analysis, loss of MAP4K5 expression was an independent prognostic factor for reduced overall survival with a hazard ratio of 2.05 (95% confidence interval: 1.17–3.56, P = 0.012). In addition, lymph node metastasis and positive resection margin were also independent predictors for reduced survival, P = 0.013 and P = 0.012 respectively, in multivariate analysis (Table 3). No significant correlation between MAP4K5 expression and other clinicopathologic features, including gender, age, tumor size, differentiation, lymph node and resection margin status, was observed in our patient population (P>0.05, Table 2)

**Table 3:** Multivariate analysis of overall survival in patients with stage II pancreatic ductal adenocarcinoma.

Variables		*N*	HR^a^ (95% CI^a^)	P value
Differentiation				
	Well to moderate	76	1.00	
	Poor	29	1.01 (0.60–1.71)	0.97
Lymph node status (stage)				
	Negative (IIA)	20	1.00	
	Positive (IIB)	85	2.30 (1.19–4.44)	0.013
Margin status				
	Negative	89	1.00	
	Positive	16	2.22 (1.20–4.13)	0.012
MAP4K5 expression				
	High	81	1.00	
	Low	24	2.05 (1.17–3.56)	0.012

^a^Abbreviations: HR, hazard ratio; CI, confidence interval.

## Discussion

MAP4K5 plays an important role in regulating a range of cellular responses of hematopoietic cells to environmental stimuli, such as TNFα through stress-activated protein kinases (SAPKs) and JNK pathways [24–26]. MAP4K5 has also been shown to be involved in both the canonical and non-canonical Wnt signaling in B lymphocytes [27]. However, its functions in human malignancies have not been studied. In this study, we showed that MAP4K5 expression was decreased or lost in 77.1% of PDAC. In contrast, MAP4K5 was expressed at high level in the normal pancreatic ductal cells in 100% of the matched non-neoplastic pancreas samples. Similarly, loss of MAP4K5 protein expression was observed in majority of human pancreatic cancer cell lines compared to HPDE cells, immortalized normal pancreatic ductal cells. More important, we demonstrated that loss of MAP4K5 protein expression was associated with reduced overall survival and was an independent prognosticator for overall survival in patients who underwent resection for PDAC by multivariate analysis. Our data suggested, for the first time, that loss of MAP4K5 expression might play a role in the progression of pancreatic cancer. Our findings were consistent with recent studies, which showed that HPK1, another member of the GCK subfamily of the mammalian Ste20-like serine/threonine kinase family, functions as a tumor suppressor both in pancreatic cancer and lung cancer [12, 13, 28]. On the other hand, MAP4K4 has been shown to be overexpressed in pancreatic cancer and other types of malignancies. Overexpression of MAP4K4 is a poor prognostic factor for both overall and disease-free survival in patients with pancreatic cancer [16]. These data suggest a divergent role of MAP4K family in cancer. Previous studies showed that targeted degradation of HPK1 in pancreatic cancer by the CUL7/Fbxw8 ubiquitin ligase involves HPK1 autophosphorylation. Activation and autophosphorylation of HPK1 form a negative-feedback loop to restrain HPK1 activity, which regulates cell cycle progression and cell proliferation in pancreatic cancer [12, 13]. It is possible that the 26S proteasome pathway may play a role in the loss of MAP4K5 expression in human malignancies. However, the molecular mechanisms leading to the loss of MAP4K5 expression in pancreatic cancer need further investigation.

The WNT/β-catenin signaling pathway plays a pivotal role in maintaining the epithelial cell phenotype and cell-cell junctions. Dysregulation of this pathway will lead to EMT, which has been shown to be involved in the development, progression, and chemotherapy resistance of pancreatic cancer [5, 6]. In light of previous findings that MAP4K5 is involved in the regulation of both the canonical and non-canonical Wnt signaling [27], we examined the functions and correlations between MAP4K5 and EMT in pancreatic cancer. We found a statistically significant association between the loss of MAP4K5 expression and the loss of E-cadherin expression, a signature immunophenotypic marker for EMT, in two large independent patient cohorts, the cohort from our institution and the TCGA cohort. In addition, we observed the same correlation between the loss of MAP4K5 expression and the loss of E-cadherin expression both at the protein and mRNA levels in multiple pancreatic cancer cell lines. Furthermore, knockdown MAP4K5 in pancreatic cancer cell lines led to decreased CDH1 mRNA expression. Therefore MAP4K5 may play an important role in regulating the expression of E-cadherin and EMT in pancreatic cancer. Our data provides a link between the loss of MAP4K5 and EMT in pancreatic cancer and may suggest that MAP4K5 signaling represents a potential therapeutic target for pancreatic cancer.

The regimens of FOLFIRINOX and Gemcitabine/Nab-paclitaxel have been shown to have improved efficacy in treating patients with metastatic pancreatic cancer compared to gemcitabine alone [29, 30]. Using a comprehensive proteomic approach, Capello *et al*. recently identified CES2 as a potential predictive marker for pancreatic cancer patients receiving neoadjuvant FOLFIRINOX [21]. They showed that high levels of CES2 expression decrease the half maximal inhibitory concentration (IC50) of irinotecan and increase the efficacy of the FOLFIRINOX regimen [21]. In this study, we found that loss of MAP4K5 expression is significantly associated with low levels of CES2 expression in our patient population. Although we did not find a significant correlation between MAP4K5 mRNA and CSE2 mRNA in TCGA population (data not shown), our findings suggest that loss of MAP4K5 may be involved in chemotherapy resistance of pancreatic cancer, especially to regimens that contain irinotecan in the backbone.

In summary, MAP4K5 protein is expressed at high levels specifically in the pancreatic ductal cells of normal pancreas. Loss of MAP4K5 expression is present in majority of pancreatic ductal adenocarcinomas and is an independent poor prognostic factor for patients with stage II PDAC. The strong associations between the loss of MAP4K5 expression and loss of E-cadherin or reduced CES2 expression may suggest an important role of MAP4K5 in epithelial-to-mesenchymal transition, chemotherapy resistance and tumor progression in pancreatic cancer. Thus, targeting impaired MAP4K5 signaling may represent a new therapeutic strategy for pancreatic cancer treatment.
